# Supplementary material for: Determinants and predictors of mental health during and after COVID-19 lockdown among university students in Malaysia
Source: PLoS One. 2023 Jan 20;18(1):e0280562. doi: 10.1371/journal.pone.0280562 (PMC9858015; doi:10.1371/journal.pone.0280562)
Supplement: Attachment — Submitted filename: Rebuttal Letter.docx [file pone.0280562.s002.docx]

**Rebuttal letter in response to the academic editor(s) and reviewer(s) comments**

Dear Academic Editor & Reviewers,

We appreciate you for your precious time in reviewing our manuscript and providing the constructive feedback. We sincerely thank you for your thorough review and for the excellent suggestions that we received. We firmly believe that the academic editors and reviewers’ suggestions have significantly improved the quality of this manuscript. The authors welcome further constructive comments if any.

We have made a concerted effort and tried our best to adequately respond to each comment made by you to amend the relevant sections of our manuscript. We do hope that the academic editor and reviewers’ find this manuscript acceptable for publication.

Below is the authors response to comments made by academic editor and reviewers. Areas highlighted in turquoise are the suggestions that we accepted and included in the manuscript, while those in red are not included in the manuscript. Our response to your comments is highlighted in yellow colour.

**Comments made by Academic reviewer**

I would encourage the authors to consider some other papers on university students' mental health:

Sharma, R., Bansal, P., Chhabra, M., Bansal, C., & Arora, M. (2021). Severe acute respiratory syndrome coronavirus-2-associated perceived stress and anxiety among Indian medical students: A cross-sectional study. Asian Journal of Social Health and Behavior, 4, 98-104.

Ahorsu, D. K., Pramukti, I., Strong C., Wnag, H.-W., Griffiths, M. D., Lin, C.-Y., Ko, N.-Y. (2021). COVID-19-related variables and its association with anxiety and suicidal ideation: Differences between international and local university students in Taiwan. Psychology Research and Behavior Management, 14, 1857-1866.

Nayan, M. I., Uddin, M. S., Hossain, M. I., Alam, M. M., Zinnia, M. A., Haq, I., Rahman, M. M., Ria, R., Haq Methun, M. I. (2022). Comparison of the performance of machine learning-based algorithms for predicting depression and anxiety among University Students in Bangladesh: A result of the first wave of the COVID-19 pandemic. Asian Journal of Social Health and Behavior, 5, 75-84.

Pramukti, I., Strong, C., Sitthimongkol, Y., Setiawan, A., Pandin M. G. R., Yen, C.-F., Lin, C.-Y., Griffiths, M. D., Ko, N.-Y. (2020). Anxiety and suicidal thoughts during the COVID-19 pandemic: A cross-country comparison among Indonesian, Taiwanese, and Thai university students. Journal of Medical Internet Research, 22(12), e24487.

Also, it would be good if the authors can have some references to indicate effective programs in improving university students' mental health.

Kadian, S., Joseph, J., Pal, S., & Devi, R. (2022). Brief resilience interventions for mental health among college students: Randomized controlled trial. Asian Journal of Social Health and Behavior, 5, 131-137.

**Authors Response**

We have added all the necessary information both on mental health of university students during COVID-19 in the introduction section and effective intervention strategies to improve the same in the discussion section citing the references suggested by academic editor, except the study conducted by Ahorsu, D. K., et al., as we believe this reference is not relevant to our inclusion criteria.

**Comments made by Reviewer 1**

First, when throughout the Introduction section, the authors correctly stressed the importance and complexity of transition into adulthood of university students, the impact of brain changes in early-onset individuals with mood disorders, particularly among young adolescents for their possible effects on childhood negative experiences, might be briefly mentioned. Importantly, more abnormalities have been documented in the brains of adolescents with bipolar depression than unipolar depression. Therefore, given the above information, my suggestion is to include the following manuscripts (PMID: 25212880; 35645550; 33417221).

In addition, the authors might further mention the consequences of Covid-19 related lockdown on lifestyle habits and behavioral risk factors. Importantly, the impact of COVID-19 lockdown on physical, mental, and social wellbeing of elderly and fragile populations in specific countries such as Italy cannot be ignored. The multi-disciplinary competencies together with appropriate funding and access to rich data sources may allow to fulfill interesting research objectives. Thus, according to this background, the following articles (PMID: 32701921; 35886439) may be cited within the main text. Moreover, as the most relevant aims/objectives of the present review paper have been reported extensively, the main hypotheses underlying this study should be reported in a similarly detailed manner. In addition, the main rationale according to which the study has been conducted and criteria based on which the online survey has been proposed and participants have been recruited need to be comprehensively described for the general readership. Moreover, the most relevant outcome measures need to be described more succinctly for the general readership.

The authors could immediately present and discuss, in the first lines of the Discussion section, the most relevant study conclusive remarks of this paper instead of focusing on the most relevant aims/objectives of the present study that should have been adequately stressed elsewhere within the main text. In addition, the most relevant shortcomings of this paper need to be extensively reported as the main caveats (e.g., the retrospective nature of the study) have been only partially described. Finally, what is the take-home message of this manuscript? While the authors reported that during lockdown, students from low-income family have higher anxiety than the higher income, and inactive students were less likely to be anxious than active students, whilst female students were more likely to be depressed when compared to male students, they could provide, in my opinion, more extensively conclusive remarks upon the main topic for the readers. What are, specifically, the main implications of these findings? How the present results may be generalized? Here, some additional information are required and might be useful for the general readership.

**Authors Response**

Brain changes among adults and adolescents diagnosed with psychological disorders is added in the introduction section with the reference support as suggested by the reviewer.

The impact of COIVID-19 lockdown on lifestyle habits and behavioural changes have been highlighted and cited in the relevant section of introduction. We believe that research findings from the Italian study (PMID: 32701921) included a wider age frame and focused on the fragile elderly population, which may not be relevant to our study, therefore, we did not include this in the introduction section.

Hypothesis is added in the last paragraph of introduction.

Methodology section is expanded as suggested by the reviewer. Details on recruitment of participants and outcomes measures together with additional changes in the methods section have been thoroughly carried out.

We have made changes in the discussion section as well. Relevant conclusive remarks are presented immediately in the first few lines of discussion section. In addition, the limitations of this study are elaborated under the limitation section and how to overcome this issue has been addressed in the future studies.

Implications of the study findings are added in the last part of the discussion and conclusion sections.

**Comments made by Reviewer 2**

In general, the manuscript needs Proofreading and exceptional attention to the consistencies from the introduction to the conclusion.

Please Provide a STROBE Statement per the cross-sectional study, prepare your manuscript according to STROBE guidelines, submit the checklist as per STROBE criteria, and attach it as a supplementary file.

Abstract
Please write the abstract as one paragraph, including all the main points for the structured abstract.

Introduction:
The paper focused on the "Determinants of Mental Health during and after COVID-19 Lockdown among University Students in Malaysia" It is hard to follow the study's main aim because there are inconsistencies between the headline, intro, findings, and discussion.

Your study focused on the effect of the COVID-19 Lockdown on University Students' Mental Health in Malaysia. However, you didn't concentrate on or highlight this main issue and what previous studies found on these issues in the introduction. Therefore, It is recommended to extend the introduction part by including how the COVID-19 Lockdown affects University Students' Mental Health and education as well globally and in Malaysia specifically; here are some studies focused on these issues, and you may find more, and later on you may use in addition to the introduction in your discussion as well to compare with your results.
- <https://doi.org/10.1371/journal.pone.0277368>
- <https://doi.org/10.3390/healthcare10101858>
- <https://doi.org/10.1016/j.sleepe.2022.100030>
- <https://doi.org/10.3389/feduc.2022.960660>
- <https://doi.org/10.3390/ejihpe12080079>

Methods:
The subsection "Role of funders" is not under the method; please move it and follow the PLOS ONE GUIDELINE.

Under the methods, please include a subsection about "Ethics and consent statement"

Results:
Please report the sociodemographic characteristics of the sample clearly, and please take note "97% were Chinese" they are Malaysian but their ethnicity is Chinese, and the study was conducted in Malaysia, and you can’t start the sentence with a number; please rewrite.

Please report the level of anxiety and depression among gender and other sociodemographic factors in the table and the text.

Discussion:

In the discussion, please highlight the theoretical and practical implications.

**Authors Response**

We have proofread the paper and tried our best to overcome the inconsistencies throughout the manuscript to improve its overall quality.

STROBE statement has been included in the methodology section and is attached as a supplementary file.

Abstract is amended to a single paragraph.

Introduction section has been extended to include how COVID-19 has affected the mental health and education of university students with the relevant reference support.

We did not include the reference no. 2 and 3 suggested by second reviewer in the revised manuscript. We believe these 2 are not relevant to our study.

Role of funders is removed from the methods section and is included as a separate section after the conclusion of study.

Subsection under the methods about ethics and consent statement is added.

Sociodemographic characteristics of the participants has been elaborated under the result section. We also included the description of depression and anxiety level based on gender under the subsection “demographics”, but not in the demographic table, as this information is already added in the table 3. We believe it will be redundant if we add gender based depression and anxiety to demographic table 1.

Practical and theoretical implications is added in the discussion section with reference support.

Below table shows the reference papers that have been included in the manuscript.

|  | **Ref Paper** | Comment/ Remark |
| --- | --- | --- |
| 1 | PMID: 25212880 | Added  **Gianluca S,** 2014.  Brain changes in early-onset bipolar and unipolar depressive disorders: a systematic review in children and adolescents |
| 2 | PMID: 35645550 | Added  **Anne I,** 2022.  College student mental health: Understanding changes in psychological symptoms in the context of the COVID-19 pandemic in the United States |
| 3 | PMID: 33417221 | Added  **Dolatshahi M**, 2021. Pathophysiological Clues to How the Emergent SARS-CoV-2 Can Potentially Increase the Susceptibility to Neurodegeneration |
| 4 | PMID: 32701921 | Not added  Odone A, 2022.  COVID-19 lockdown impact on lifestyle habits of Italian adults  Representative sample of adults aged 18-74 years**.** |
| 5 | PMID: 35886439 | Added  **Patrono A**, 2022.  COVID-19 Aftermath: Exploring the Mental Health Emergency among Students at a Northern Italian University  (in discussion as well as intro) |
| 6 | https://doi.org/10.1371/journal.pone.0277368 | Added  **Aljaberi MA**, 2022  A cross-sectional study on the impact of the COVID-19 pandemic on psychological outcomes: Multiple indicators and multiple causes modeling |
| 7 | https://doi.org/10.3390/healthcare10101858 | Not added  Aljaberi MA 2022, Rasch Modeling and Multilevel Confirmatory Factor Analysis  Irrelevant |
| 8 | https://doi.org/10.1016/j.sleepe.2022.100030 | Not added  El Abiddine 2022, Mediated effects of insomnia in the association between problematic social media use and subjective well-being  Does not align with our research objectives |
| 9 | https://doi.org/10.3389/feduc.2022.960660 | Added  **Al-Tammemi AB,** 2022  A qualitative exploration of university students’ perspectives on distance education in Jordan: An application of Moore’s theory of transactional distance |
| 10 | https://doi.org/10.3390/ejihpe12080079 | Added  **Mohammed LA,** 2022  Exploring Factors Affecting Graduate Students’ Satisfaction toward E-Learning in the Era of the COVID-19 Crisis |
| 11 | Sharma, R., Bansal, P., Chhabra, M., Bansal, C., & Arora, M. (2021). Severe acute respiratory syndrome coronavirus-2-associated perceived stress and anxiety among Indian medical students: A cross-sectional study. Asian Journal of Social Health and Behavior, 4, 98-104. | Added |
| 12 | Ahorsu, D. K., Pramukti, I., Strong C., Wnag, H.-W., Griffiths, M. D., Lin, C.-Y., Ko, N.-Y. (2021). COVID-19-related variables and its association with anxiety and suicidal ideation: Differences between international and local university students in Taiwan. Psychology Research and Behavior Management, 14, 1857-1866. | Not added  International students vs. locals.  Not relevant to our inclusion criteria. |
| 13 | Nayan, M. I., Uddin, M. S., Hossain, M. I., Alam, M. M., Zinnia, M. A., Haq, I., Rahman, M. M., Ria, R., Haq Methun, M. I. (2022). Comparison of the performance of machine learning-based algorithms for predicting depression and anxiety among University Students in Bangladesh: A result of the first wave of the COVID-19 pandemic. Asian Journal of Social Health and Behavior, 5, 75-84. | Added |
| 14 | Pramukti, I., Strong, C., Sitthimongkol, Y., Setiawan, A., Pandin M. G. R., Yen, C.-F., Lin, C.-Y., Griffiths, M. D., Ko, N.-Y. (2020). Anxiety and suicidal thoughts during the COVID-19 pandemic: A cross-country comparison among Indonesian, Taiwanese, and Thai university students. Journal of Medical Internet Research, 22(12), e24487 | Added |
| 15 | Kadian, S., Joseph, J., Pal, S., & Devi, R. (2022). Brief resilience interventions for mental health among college students: Randomized controlled trial. Asian Journal of Social Health and Behavior, 5, 131-137 | Added |

Warm Regards,

Sincerely,

Imtiyaz Ali Mir

Senior Lecturer, Faculty of Medicine & Health Sciences,

Universiti Tunku Abdul Rahman
